# Articles You Might Have Missed

**DOI:** 10.1007/s13181-025-01107-3

**Published:** 2025-12-02

**Authors:** Kevin Brandecker, Kayla Kendric, Christopher Raciti, Paul F. Ehlers

**Affiliations:** 1 Department of Emergency Medicine, University of California, San Francisco, San Francisco, CA USA; 2 California Poison Control System, San Francisco Division, San Francisco, CA USA; 3 Zuckerberg San Francisco General Hospital and Trauma Center, UCSF Department of Emergency Medicine, 1001 Potrero Avenue, Bldg. 10, Ward 12, Room #1207, San Francisco, CA 94143 USA

**Keywords:** Opioid use disorder, Immune-related adverse effects, Pneumonitis, Cannabidiol, Drug induced liver injury, BTMPS

## Article #1: Naloxone Distribution Programs

Fischer LS, Asher A, Stein R, et al.: Effectiveness of naloxone distribution in community settings to reduce opioid overdose deaths among people who use drugs: a systematic review and meta-analysis. *BMC Public Health*. 2025;25:1135. 10.1186/s12889-025-22210-8. 

### Background

Over 111,000 overdose deaths occurred in the U.S. during the 12 months ending July 2023, with over three-quarters involving opioids. Naloxone can reverse opioid overdoses but remains underutilized in community settings. Community-based overdose education and naloxone distribution (OEND) programs have expanded but still face barriers such as stigma, cost, and limited access.

### Research Question

How effective are community-based naloxone distribution programs at reducing opioid overdose deaths among people who use drugs and other laypersons in non-clinical settings?

### Methods

Systematic review and meta-analysis of peer-reviewed studies (2003–2022), divided into two timeframes: Group 1 (2003–2018) and Group 2 (2018–2022). Studies included reported individual-level survival outcomes following naloxone administration in community-based OEND programs. Data were analyzed by population group, naloxone route, dose, training duration, and geographic location.

### Results

In the first group of 41 studies conducted between 2003 and 2018, overall survival after naloxone administration in community settings was 97.3%. When stratified by population, survival rates were highest among people who use drugs (PWUD) (98.3%), followed by family or community members (95.0%), and law enforcement personnel (92.4%). The second group of studies (2018–2022) demonstrated similarly high effectiveness, even as illicit fentanyl became increasingly prevalent, confirming that naloxone distribution remained effective despite the more potent opioid landscape. At the community level, areas where overdose education and naloxone distribution (OEND) programs were implemented saw reductions in overdose mortality of up to 46%, underscoring not only the individual-level benefit but also the broader population impact of these interventions.

### Conclusion

Community-based naloxone distribution programs are highly effective at reducing opioid overdose deaths, with sustained high survival rates even amid the rise of fentanyl. The findings support broader implementation and access to naloxone in community settings.

### Critique

Strengths include a large number of studies, robust meta-analytic methods, and real-world applicability. Limitations include reliance on passive follow-up, potential publication bias, lack of control groups, and limited post-resuscitation outcome data. Many studies were conducted by program implementers, introducing potential bias.

### Implication for Toxicologists

Toxicologists should advocate for widespread naloxone access and support OEND efforts, especially among people who use drugs PWUD. They can play a role in education, policy development, and removing barriers to community naloxone distribution, particularly in settings affected by fentanyl and polysubstance use.

## Article #2: Immune Checkpoint Inhibitor-Associated Pneumonitis

Li C, Faiz SA, Boysen-Osborn M, Sheshadri A, et al.: Immune checkpoint inhibitor-associated pneumonitis: a narrative review. *Western Journal of Emergency Medicine*. 2025;26:210–218. 10.5811/westjem.20305.

### Background

Immune checkpoint inhibitors (ICI) have been shown to improve survival rates in multiple types of malignancies when used as a single agent or in conjunction with other chemotherapeutics. This has led to ICI being widely used as a treatment modality. However, these medications come with a unique set of immune-related adverse effects (irAE) and drug toxicities that can be easily missed. One of the most common ICI-associated adverse events is pneumonitis, with delay in diagnosis leading to worsening outcomes.

### Research Question

What are common clinical presentations and risk factors for immune checkpoint inhibitor pneumonitis, and what is the approach to diagnosis and management in the Emergency Department?

### Methods

This is a narrative review focused on ICI-associated pneumonitis, exploring the diagnostic approach and management in the emergency department.

### Results

ICI pneumonitis usually presents 6–12 weeks after initiation of ICI but can be seen sooner in those with underlying pulmonary diseases or primary pulmonary malignancy. Patients often present with fatigue, dyspnea, cough, and hypoxia. They may also develop other irAEs, like skin lesions, colitis, or endocrine complications.

Patients receiving PD-1 therapy are at a higher risk of developing pneumonitis, specifically those with advanced non-small cell lung cancer. This risk can be further increased if they are being co-administered with PD-L1 inhibitors. Underlying interstitial lung disease, tobacco use, or other autoimmune diseases also cause patients to have an increased risk. Pneumonitis due to ICI is a clinical diagnosis without specific radiographic findings, and workup centers on ruling out other causes. Management focuses on cessation of treatment with ICI and may also require treatment with steroids or other immunosuppressives. Ultimately, patients may be reinitiated on ICI with close monitoring for recurrence given higher risk for toxicity.

### Conclusion

ICI have had a dramatic impact on survival in many different cancer types and are therefore frequently prescribed. Oncology patients are commonly seen in the emergency department, given their chronic medical conditions, immunosuppressed state, and risk for medication side effects. The key step is to consider ICI as a cause of pneumonitis so care can be coordinated with oncologists and other specialists. Physicians need to maintain a high index of suspicion for irAEs, as they can mimic other common causes of dyspnea or hypoxia in the oncology patient.

### Critique

Unlike a systematic review, there was not an exhaustive search of the medical literature, and there is no attempt at quantitative meta-analysis.

### Implication for Toxicologists

While oncologists closely monitor their patients for toxicity that comes with many of the medications that they use, these patients often present to the emergency department. Medical toxicologists should understand the pathophysiology, diagnosis, and management of irAEs and specifically pneumonitis.

## Article #3: Cannabidiol and Liver Enzyme Elevations

Florian J, Salcedo P, Burkhart K, Shah A, et al.: Cannabidiol and liver enzyme level elevations in healthy adults: a randomized clinical trial. *JAMA Internal Medicine*. 2025;185:1070–8. 10.1001/jamainternmed.2025.2366.

### Background

The use of unregulated CBD products has grown in recent years, yet limited safety data exist regarding doses typically consumed by users of these products. Reports of liver enzyme elevations at relatively high prescription doses of CBD highlight the potential for drug-induced liver injury.

### Research Question

What are the effects of daily cannabidiol (CBD) use in healthy adults at doses representative of consumer use on liver enzyme levels and endocrine hormone levels?

### Methods

This was a randomized, double-blind, placebo-controlled trial evaluating the effects of daily CBD use on liver enzymes and endocrine measures in healthy adults at doses within the range commonly used in commercially-available products. Participants (*n* = 201) received oral CBD at 2.5 mg/kg twice daily for 28 days. Chemistry and hematology labs were measured on days − 1, 1, 7, 14, 21, 28, 29, and 35, and endocrine assessments (total testosterone and inhibin B in males; TSH, total T3, and free T4 in all participants) were measured on days 1 and 29. The primary outcome was the percentage of participants with ALT or AST elevations greater than three times the upper limit of normal (ULN). Secondary outcomes included liver safety withdrawal criteria for potential drug-induced liver injury and changes in endocrine parameters compared with placebo.

### Results

Participants were randomized to receive CBD (*n* = 151) or placebo (*n* = 50). After 4 weeks of dosing, 8 participants in the CBD group (5.6% [95% CI, 1.8%–9.3%]) and none in the placebo group (0%; [CI: 0 – 7.6%]) experienced ALT elevations greater than 3 times the upper limit of normal. Seven participants (4.9% [95% CI, 1.3%–8.4%]) in the CBD group met withdrawal criteria for potential DILI. No cases progressed to clinically apparent liver injury.

There were no substantial differences between CBD and placebo groups in endocrine measures, including testosterone and inhibin B in men or thyroid-stimulating hormone, total triiodothyronine, and free thyroxine in all participants. Overall, 52 participants (26%) experienced adverse events, occurring more often in the CBD group (43 [29%]) than placebo (9 [18%]). The most common adverse events with CBD were hepatic enzyme elevation (11%), eosinophilia (9%), somnolence (8%), and diarrhea (8%). No serious or life-threatening adverse events were reported.

### Conclusion

Daily CBD use at doses comparable to those in marketed products was associated with a measurable risk of significant liver enzyme elevations and potential drug-induced liver injury, though cases were reversible and not clinically severe. No meaningful effects were observed on endocrine function.

### Critique

This study’s strengths include its randomized, double-blind, placebo-controlled design, relatively large sample size, and systematic monitoring of liver and endocrine function, providing safety data at doses relevant to consumer use. However, its short 28-day treatment period limits assessment of longer-term risks, and the inclusion of only healthy adults reduces generalizability to populations with comorbidities or concomitant medication use. Additionally, while elevations in liver enzymes were observed, the study was not powered to assess clinically significant outcomes.

### Implication for Toxicologists

This study provides evidence that CBD use at consumer-relevant doses can cause liver enzyme elevations. These findings may be used to educate patients and clinicians on potential risks of unregulated CBD products and guide future research into long-term safety and monitoring strategies.

## Article #4: BTMPS

Zhu DT, Krotulski AJ, Palamar JJ.: The rapid spread of a novel adulterant in the US illicit drug supply—BTMPS. *JAMA Internal Medicine.* 2025;185:1057–8. 10.1001/jamainternmed.2025.2307.

### Background

Although they have decreased recently, drug overdose deaths remain a leading cause of preventable mortality in the US. The illicit drug supply undergoes significant volatility, with frequently changing adulterants. Recently, the substance bis(2,2,6,6-tetramethyl-4piperidyl) sebacate (BTMPS) has been detected. BTMPS is a hindered amine light stabilizer most commonly used as a UV protectant in plastics. Its purpose in the drug supply is not known and it was not intended or studied for use in humans. It is possible that BTMPS is being included as a bulking agent, as it is often detected with higher mass in drug samples than fentanyl. It may also stabilize or protect fentanyl from UV-degradation.

### Research Question

What is the geographic spread of and temporal trends in BTMPS adulteration in the illicit drug supply?

### Methods

Research letter, using data from the National Forensic Laboratory Information System (NFLIS) and other recently published surveys of the illicit drug supply.

### Results

BTMPS was first detected in June 2024 in fentanyl samples in Portland, Oregon and Philadelphia, Pennsylvania, but by September 2024 was found in nearly every state. 56% of fentanyl samples in Los Angeles, California and 32% in Philadelphia, Pennsylvania contained BTMPS in September 2024. National data confirmed these regional trends: NFLIS documented 1969 reports of BTMPS in 2024, with 99% occurring in the latter half of the year.

### Conclusion

BTMPS adulteration of illicit opioids has a widespread geographic distribution and its spread has been rapid. Its purpose is unknown. BTMPS’s appearance across broad geographic areas contemporaneously suggests a centralized upstream source or systemic contamination rather than an isolated local contamination.

### Critique

As a research letter, this paper is brief and finer methodological details are not present. Underlying data on BTMPS, especially in humans, are extremely scant, limiting definitive conclusions. Many of the assumptions regarding the purpose of BTMPS contamination are speculative.

### Implication for Toxicologists

BTMPS is essentially unstudied in humans, but animal studies have revealed L-type calcium channel and nicotinic acetylcholine receptor antagonism. Serious health effects such as cardiotoxicity, sedation, and pulmonary injury have been identified in animal models. Patients presenting after an opioid exposure with unexpected autonomic or circulatory effects may be suffering from BTMPS toxicity, and mass spectrometry testing of patient or drug samples should be considered. There is no known antidotal therapy for BTMPS.

